# Parliament and the Profession

**Published:** 1917-05-12

**Authors:** 


### Parliament and the Profession.

### Hospital Ships.

The Minister of Blockade (Lord Robert Cecil) stated that the Government consider that no useful purpose will be served by requesting the German Government to state what proof it requires that British hospital ships are not carrying munitions of war.

### Venereal Diseases.

Mr. Hayes Fisher informed Mr. King that leaflets containing instructions on the lines suggested by the Royal Commission were issued by the Local Government Board in December last for distribution to doctors and hospitals, and are being supplied gratis to all doctors for the use of patients suffering from venereal disease.

### Malaria.

Mr. Macpherson, in answer to a question by Mr. Cowan, said that centres for the treatment of malaria have been established in each command, and officers with experience of this disease have been placed in charge of them. Malarial cases are now being put through a course of treatment with the hope of eradicating the disease.

### Maintenance of Pauper Lunatics in Ireland.

The Chief Secretary for Ireland stated that there is no immediate prospect of an increase in the Imperial capitation grant in aid of local taxation in respect of the maintenance of pauper lunatics. The grant paid in 1916-17 was at the rate of £9 0s. 9d. per patient.

